# Loss of Raf-1 kinase inhibitor protein (RKIP) is strongly associated with high-grade tumor budding and correlates with an aggressive phenotype in pancreatic ductal adenocarcinoma (PDAC)

**DOI:** 10.1186/1479-5876-11-311

**Published:** 2013-12-14

**Authors:** Eva Karamitopoulou, Inti Zlobec, Beat Gloor, Agathi Kondi-Pafiti, Alessandro Lugli, Aurel Perren

**Affiliations:** 1 Clinical Pathology Division, University of Bern, Murtenstrasse 31, Bern, Switzerland; 2 Institute of Pathology, Translational Research Unit, University of Bern, Murtenstrasse 31, Bern, Switzerland; 3 Department of Visceral Surgery, Insel University Hospital, Bern, Switzerland; 4 Department of Pathology, Aretaieion University Hospital, University of Athens, Athens, Greece

**Keywords:** RKIP, Pancreatic cancer, Epithelial-mesenchymal transition, Tumor buds

## Abstract

**Background:**

Raf-1 kinase inhibitor protein (RKIP) has emerged as a significant metastatic suppressor in a variety of human cancers and is known to inhibit Ras/Raf/MEK/ERK signaling. By suppressing the activation of the NFkB/SNAIL circuit, RKIP can regulate the induction of epithelial-mesenchymal transition (EMT). The aim of this study was to evaluate RKIP expression and to determine its association with clinicopathological features, including EMT in form of tumor budding in pancreatic ductal adenocarcinoma (PDAC).

**Methods:**

Staining for RKIP was performed on a multipunch Tissue Microarray (TMA) of **114** well-characterized PDACs with clinico-pathological, follow-up and adjuvant therapy information. RKIP-expression was assessed separately in the main tumor body and in the tumor buds. Another 3 TMAs containing normal pancreatic tissue, precursor lesions (Pancreatic Intraepithelial Neoplasia, PanINs) and matched lymph node metastases were stained in parallel. Cut-off values were calculated by receiver operating characteristic (ROC) curve analysis.

**Results:**

We found a significant progressive loss of RKIP expression between normal pancreatic ductal epithelia (average: 74%), precursor lesions (PanINs; average: 37%), PDAC (average 20%) and lymph node metastases (average 8%, p < 0.0001). RKIP expression was significantly lower in tumor buds (average: 6%) compared to the main tumor body (average 20%; p < 0.005). RKIP loss in the tumor body was marginally associated with advanced T-stage (p = 0.0599) as well as high-grade peritumoral (p = 0.0048) and intratumoral budding (p = 0.0373). RKIP loss in the buds showed a clear association with advanced T stage (p = 0.0089).

**Conclusions:**

The progressive loss of RKIP seems to play a major role in the neoplastic transformation of pancreas, correlates with aggressive features in PDAC and is associated with the presence of EMT in form of tumor budding.

## Background

RAF kinase inhibitor protein (RKIP; also known as PEBP, for phosphatidylethanolamine-binding protein) is considered a metastasis suppressor protein, acting as an endogenous inhibitor of the Raf–mitogen-activated protein (MAP) kinase (MEK)–extracellular signal-regulated kinase (ERK) pathway by inhibiting the phosphorylation and activation of MEK by Raf-1 [1,2]. It has additionally been shown that RKIP suppresses the activation of the NFkB/SNAIL circuit [3-6]. This pathway plays an important role in the induction of epithelial mesenchymal transition (EMT) of cancer cells as one of the initial steps for metastatic spread [3-6].

A reduced RKIP expression has been shown to be associated with tumor progression and unfavourable prognosis in a variety of human malignant tumors [7-9]. In recent studies performed by our group on colorectal cancer (CRC), we could demonstrate that RKIP status, when combined with N stage and vascular invasion can provide independent prognostic information on metastatic disease [10]. In a TMA-based profiling of multi-marker phenotypes of CRC, we identified RKIP as a predictor of high grade tumor budding with a differential expression between tumor center and tumor front [11,12]. Moreover, in a geographic analysis of RKIP on whole tissue sections of CRC, we demonstrated that loss of RKIP expression in the tumor center was an independent prognostic factor and could predict the chemotherapy response [13].

Pancreatic ductal adenocarcinoma (PDAC) is a common cause of cancer death and has a dismal prognosis [14]. Most patients have advanced stage disease at presentation with a median survival of less than 1 year [15]. Therapeutic options are limited with surgical resection being the only potentially curative treatment. Classical histopathologic findings as tumor size, blood vessel or lymphatic invasion and presence of lymph node metastases constitute essential prognostic factors in pancreatic cancer with tumor stage being the most important of all [16]. The lethal nature of PDAC has been attributed to the propensity of PDAC cells to rapidly disseminate to the lymphatic system and distant organs [17]. Within this context and considering the fact that the management of PDAC remains suboptimal and that adjuvant therapy has resulted to limited progress, there is a need for additional reliable and reproducible prognostic markers that would enable better patient stratification and would provide a guide towards an individualized therapy.

Tumor budding reflects a type of diffusely infiltrative growth frequently observed in gastrointestinal carcinomas and it is defined as the presence of detached, isolated single cells or small cell clusters (up to 5 cells) scattered in the stroma at the invasive tumor front [18-25] and has been suggested to actually reflect the process of EMT [26,27]. In a previous study from our group we could show that tumor budding occurs frequently in pancreatic cancer and may be used as a parameter of tumor aggressiveness and as an indicator of unfavourable outcome, even within this group of patients with generally poor prognosis [28].

Due to our previous findings on colorectal cancer, we hypothesized that loss of RKIP may be associated with the frequent occurrence of tumor budding in pancreatic cancer and may play a role in pancreatic carcinogenesis. We therefore undertook the analysis of RKIP expression on a multi-punch TMA from a well-characterized cohort of 120 pancreatic ductal adenocarcinomas (PDACs), their precursor lesions (PanINs) and matched lymph node metastases. RKIP expression was correlated with clinicopathological data and especially with the presence of tumor budding. The REMARK guidelines were used as a basis for this biomarker study [29].

## Material and methods

### Patients and specimen characteristics

120 non-consecutive PDAC patients surgically treated between 2000 and 2010 were randomly selected. Paraffin-embedded tissue blocks of primary tumors were retrieved from the Department of Pathology, Aretaieion University Hospital, University of Athens Medical School, Greece. All histomorphological data were reviewed from the corresponding hematoxylin and eosin (H&E) stained slides, while clinical data were obtained from chart reports. Clinicopathological information for all patients included age, gender, tumor diameter, number of positive lymph nodes and total number of lymph nodes harvested, TNM stage (7th Edition), perineural, as well as blood vessel and lymphatic invasion and resection margin status (R-status). Information on post-operative therapy was available for all patients. The use of this material was approved by the local ethics committees of the University of Athens and University of Bern.

### Assay methods

#### *a. Construction of tissue microarray (TMA)*

For each patient, the hematoxylin and eosin slides of the primary tumor from the corresponding whole tissue sections were evaluated and representative areas of the tissue were marked using a felt-tip pen for easy detection. To exclude bias because of possible tumor heterogeneity, each patient had 4 tumor punches (2 tumor center + 2 tumor front) taken from formalin-fixed, paraffin-embedded blocks using a tissue cylinder with a diameter of 0.6 mm that were subsequently transferred into 1 recipient paraffin block (3 × 2.5 cm) using a homemade semiautomated tissue arrayer. Tissues were obtained from the tumor center and the invasive tumor front so that each patient had at least 4 tumor punches included on this array (total of 480 punches). Three additional one-punch TMAs were constructed including normal pancreatic tissue (147 punches), precursor lesions (PanINs; 123 punches) and matched lymph node metastases (94 punches).

#### *b. Immunohistochemistry*

TMA blocks were cut at 4 μm and immunostained for pan-cytokeratin AE1/AE3 (Monosan; 1:100), that served to highlight areas of tumor budding and RKIP (Merck Millipore, Billerica, MA; 1:100). Sections were de-waxed and re-hydrated in dH2O. RKIP staining was performed using a Bond Max Autostainer from Leica Microsystem (Wetzlar, Germany) with antigen retrieval performed in citrate buffer at 100 for 20 min. Normal pancreatic tissue and nerve tissue served as an internal positive control. Negative control was obtained by omitting the primary antibody. Whole tissue sections of 5 randomly selected cases were stained in parallel as validation of the TMA results.

#### *c. Assessment of tumor budding*

**Figure 1:**
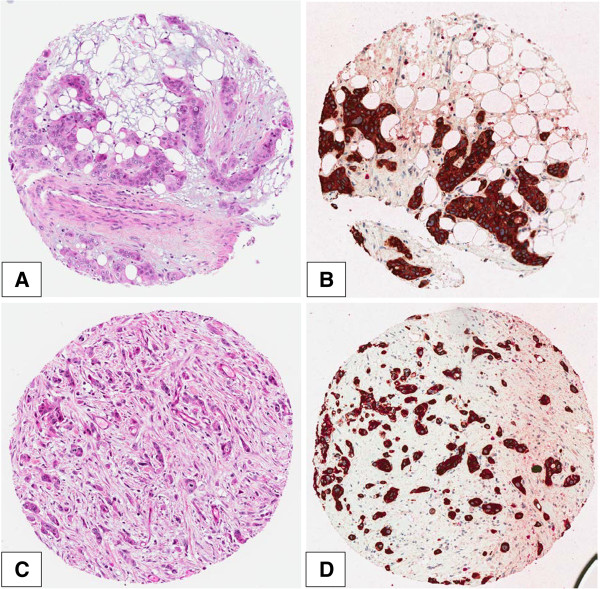
AE1/AE3 (Pancytokeratin) and H&E staining in PDAC to demonstrate the presence of tumor budding (x200): A (H&E) and B (Pancytokeratin): Low-grade tumor budding; C (H&E) and D (Pancytokeratin): High-grade tumor budding.

Tumor budding was defined as detached single cells or clusters of < 5 cells. Cases were evaluated for tumor budding using a 10-in-10 approach [30]. Briefly, whole tissue sections of each case underwent immunohistochemistry for AE1/AE3 (pan-cytokeratin) staining. The 10 densest hot-spots of tumor budding were evaluated at high-magnification (40x, 0.55 mm2) and counted. The average number of buds per case was obtained. Although tumor budding is described to occur mostly at the invasive front of cancers (peritumoral budding), in our PDAC series we frequently observed the presence of buds within the main tumor body as well (intratumoral budding). Using a receiver operating characteristic (ROC) curve approach, a cut-off score of 10 buds on average was identified as most discriminatory for survival. Cases with an average of >10 buds were classified as “high-grade” budders; those with ≤10 buds were assigned as “low-grade” budders [30] (Figure 1).

#### *d. Assessment of RKIP staining*

Immunohistochemistry was evaluated by estimating visually the percentage of positive cells per tissue microarray punch in 5% intervals (0%, 5%, …, 100%). In the case of multiple tumor punches per localization, the average protein expression was calculated across all punches from the same localization. The end result was that each patient had a final protein expression score for the main tumor body, the tumor buds, the matched lymph node metastases, the precursor lesions (PanINs) and the normal pancreatic tissue (ductal epithelia). Evaluation was performed blinded to clinical endpoints.

#### *e. Statistical analysis*

In order to determine a valid cut-off score for RKIP expression (low/high), receiver operating characteristic (ROC) curve analysis was performed, using the endpoint of tumor budding. A threshold value of 10% was identified. Association of RKIP expression with categorical clinicopathological features was performed using the Chi-Square test with continuity correction and the Fisher’s Exact tests; for continuous variables such as age and tumor size, the non-parametric Wilcoxon’s Rank Sum test was used. For matched analyses, the Wilcoxon’s Signed Rank test for pairs and the Cochran-Mantel-Haenszel test for three of more groups were used. Logistic regression analysis was used to determine the odds ratio (OR) and 95%CI for loss of RKIP expression with certain clinicopathological features. Missing data were few and were assumed to be missing at random. No imputation for missing values was performed. Univariate survival time analysis was performed using the log-rank test and differences plotted using Kaplan-Meier curves. P-values <0.05 were considered statistically significant. Correction for multiple hypothesis testing was not carried out [31]. Analyses were carried out using SAS (V9.2; The SAS Institute, Cary, NC).

## Results

### Patient characteristics and RKIP expression (Table 1)

One hundred and twenty patients with PDAC were included in this study. RKIP expression could be successfully evaluated in 114 and included 33 cases (29%) with low-expression and 81 cases (71.1%) with high-expression. Median overall survival (OS) for the cohort of 114 patients was 11 months (95%CI: 10–13), while the median disease-free interval (DFI) was 5.0 months (95%CI: 4–6). Patient characteristics are outlined in Table 1.

**Table 1:** Association of RKIP expression in main tumor with clinicopathological features (n = 114)

**Feature**		**Total N (%)**	**Frequency N (%)**		**P-value**
			**Low (n = 33; 29.0%)**	**High (n = 81; 71.1%)**	
Sex (n = 113)	Female	52 (46.0)	17 (53.1)	35 (43.2)	0.4573
	Male	61 (54.0)	15 (46.9)	46 (56.8)	
Age at diagnosis (yrs) (n = 112)	Median (min, max)	65 (35–84)	65 (42–83)	65 (35–84)	0.8265
Tumor size (cm) (n = 109)	Median (min, max)	3.0 (1.2-10)	3.0 (1.3-6.5)	3.0 (1.2-10)	0.5498
pT classification (n = 112)	pT1-2	10 (8.9)	0 (0.0)	10 (12.5)	0.0599°
	pT3-4	102 (91.1)	32 (100.0)	70 (87.5)	
Tumor grade (n = 114)	G1-2	16 (14.0)	2 (6.1)	14 (17.3)	0.205
	G3	98 (86.0)	31 (93.9)	67 (82.7)	
pN classification (n = 112)	pN0	20 (17.9)	5 (15.6)	15 (18.8)	0.9068
	pN1-2	92 (82.1)	27 (84.4)	65 (81.3)	
pM classification (n = 112)	pM0	103 (92.0)	28 (87.5)	75 (93.8)	0.4749
	pM1	9 (8.0)	4 (12.5)	5 (6.3)	
Pn classification (n = 110)	Pn0	1 (0.9)	0 (0.0)	1 (1.3)	1.0
	Pn1	109 (99.1)	32 (100.0)	77 (98.7)	
Lymphatic invasion (n = 112)	Negative	21 (18.8)	4 (12.5)	17 (21.3)	0.4215
	Positive	91 (81.3)	28 (87.5)	63 (78.8)	
Venous invasion (n = 112)	Negative	88 (78.6)	24 (75.0)	64 (80.0)	0.7431
	Positive	24 (21.4)	8 (25.0)	16 (20.0)	
R classification (n = 111)	R0	78 (70.3)	21 (65.6)	57 (72.2)	0.6511
	R1	33 (29.7)	11 (34.4)	22 (27.9)	
Adjuvant chemo (n = 105)	Untreated	3 (2.9)	0 (0.0)	3 (4.1)	0.6204
	Treated	102 (97.1)	31 (100.0)	71 (96.0)	
Tumor budding 10-in-10	Low-grade	33 (30.8)	3 (10.3)	30 (38.5)	0.0048 *
(n = 107)	High-grade	77 (69.2)	26 (89.7)	48 (61.5)	
Intratumoral budding	Negative/Low	48 (42.9)	9 (27.3)	39 (49.4)	0.0373 **
(n = 112)	Medium/High	64 (57.1)	24 (72.7)	40 (50.6)	

Cutoff 10% (ROC cutoff value).

*Odds ratio estimate for high-grade tumor budding predicted by loss of RKIP is OR (95%CI): 5.42 (1.51-19.5).

** Odds ratio estimate of medium/high grade budding predicted by loss of RKIP is OR (95%CI): 2.6 (1.07-6.29).

### RKIP expression normal-PanIN-carcinoma-metastasis sequence

**Figure 2:**
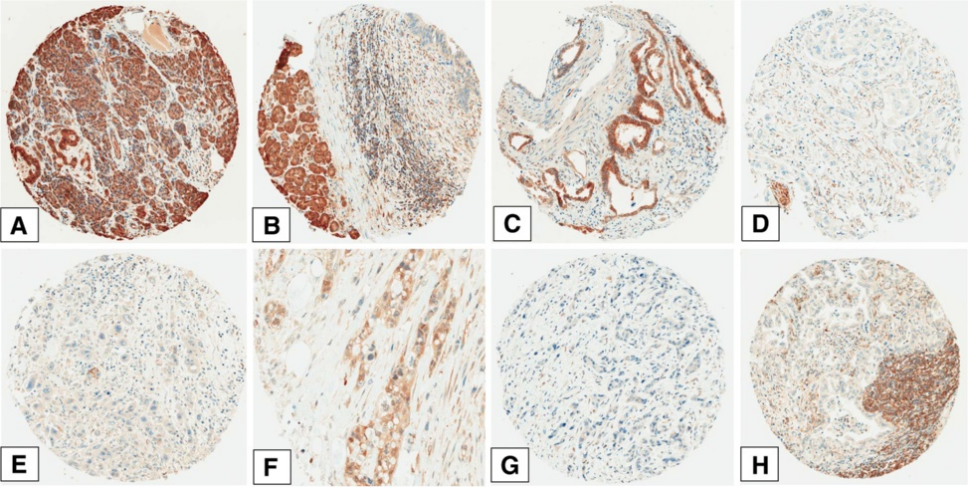
**Representative examples of RKIP staining: A: normal pancreatic tissue with strong RKIP expression (x200); B: An example of PanIN (upper right) with reduced RKIP expression compared to the normal tissue (left side of the image; x200); C: An example of PDAC with low-grade budding and retained RKIP expression (x200); D: PDAC with loss of RKIP expression.** A peripheral nerve (lower left) with strong RKIP staining (x200); **E**: PDAC with many tumor buds and loss of RKIP expression (x200); **F**: an example of PDAC with loss of RKIP expression in the buds (arrows) compared to the main tumor body (x400); **G**: PDAC with high-grade tumor budding and loss of RKIP expression (x200); **H**: Lymph node metastasis of PDAC with loss of RKIP expression (x200).

A panel of representative examples of RKIP expression can be seen in Figure 2, including examples of normal pancreatic tissue, PanINs and cancer tissue with and without budding.

**Figure 3:**
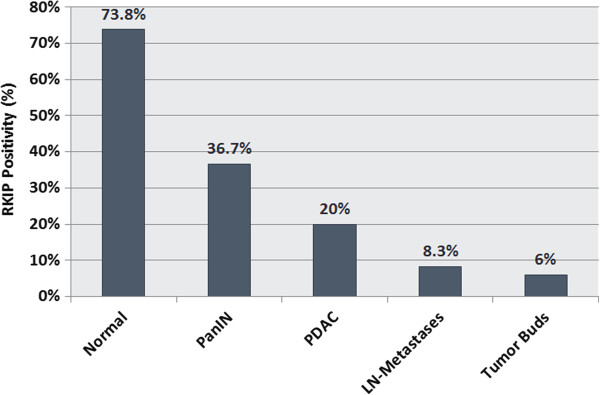
Distribution of average RKIP expression according to Histology.

**Figure 4:**
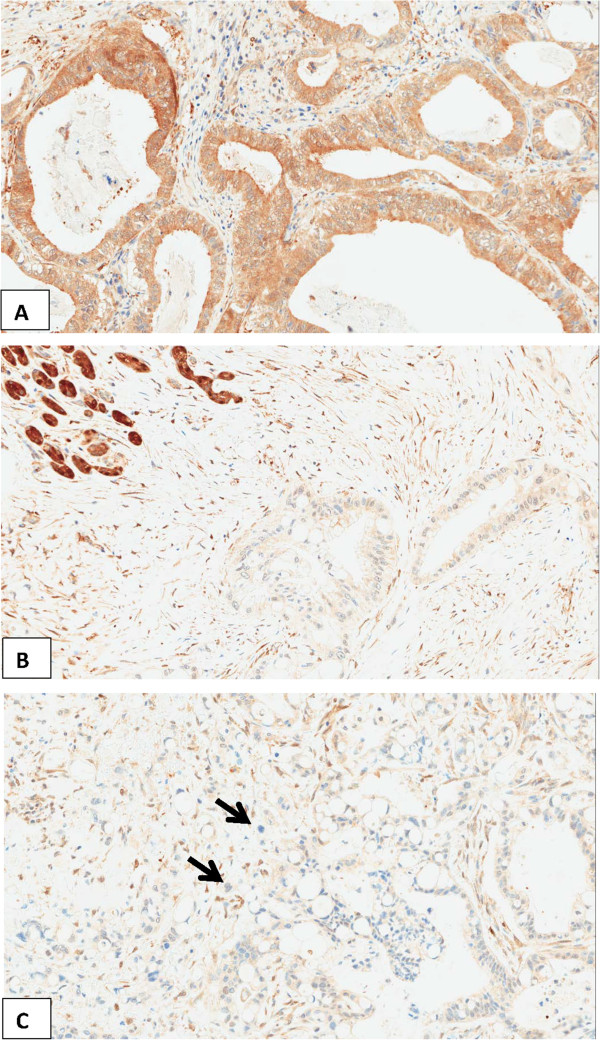
**Examples of whole tissue sections of PDAC cases immunostained for RKIP (x200). A**: positive immunostaining for RKIP in PDAC. Note the absence of tumor buds. **B**: Normal pancreatic tissue (upper left) with retained RKIP-positivity. Reduced RKIPexpression in the adjacent PDAC. **C**: Reduced RKIP-expression in PDAC and absent RKIPexpression in the tumor buds (arrows).

RKIP expression was restricted to the cytoplasm. A significant and striking progressive loss of RKIP expression was found from the normal pancreatic tissue including normal ductal epithelia (73.8%) to PanIN (36.7%), then to carcinoma (20%) and finally to lymph node metastases (8.3%) (p < 0.0001). Interestingly, within tumor budding cells of the primary carcinomas, RKIP expression was nearly absent (average expression 6%) (Figure 3). Using a matched pairs analysis (signed rank test) to test the difference between RKIP expression in the tumor and corresponding tumor buds, a statistical significant loss of expression (p < 0.0001) was observed. The parallel stained whole tissue sections revealed the same findings (Figure 4).

### RKIP expression and clinicopathological data

**Figure 5:**
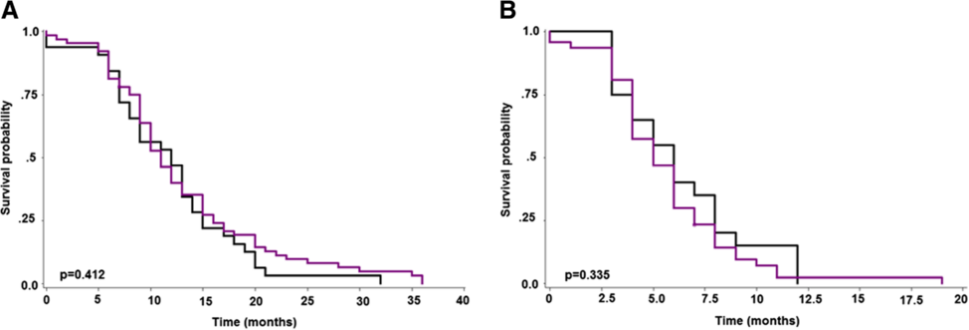
**Kaplan-Meier curves illustrating absence of survival time differences for patients with low or high RKIP expression. A)** Overall survival; **B)** Disease-free interval.

Table 1 highlights the associations between RKIP expression and clinicopathological features in PDAC. Using Fisher’s Exact test, a statistically significant association between high-grade tumor budding and loss of RKIP expression was observed (p = 0.0048). Similar results were found for intratumoral budding (p = 0.0373). In order to underline the relationship between high-grade tumor budding and loss of RKIP, logistic regression analysis was performed. OR (95%CI) for high-grade budding was 5.42 (1.51-19.5), indicating that the odds of such a phenotype were more than 5x more likely in patients with RKIP loss in comparison to those with highly expressing tumors. The sensitivity, specificity and accuracy for this finding were 0.909, 0.35, and 0.63, respectively. Similarly, the odds of intratumoral budding were 2.6 (95%CI: 1.07-6.29) times greater in patients with loss of RKIP as compared to patients with high expression of the protein. Sensitivity, specificity and accuracy were 0.812, 0.375, and 0.594, respectively. There was no association with overall survival (p = 0.412) or with disease-free interval (p = 0.335) (Figure 5).

Table 2 shows the association of RKIP expression within tumor budding cells themselves and clinicopathological features. Loss of RKIP expression within tumors buds themselves was significantly more frequent in patients with pT3-4 (p = 0.0089) carcinomas. There was no association between overall survival (p = 0.6683) or disease-free interval (p = 0.8572).

**Table 2:** Association of RKIP expression in tumor buds with clinicopathological features (n = 98)

**Feature**		**Frequency N (%)**		**P-value**
		**Low (n = 68; 69.4)**	**High (n = 30; 30.6)**	
Sex (n = 97)	Male	42 (62.7)	12 (40.0)	0.0476
	Female	25 (37.3)	18 (60.0)	
Age at diagnosis (yrs)	Median (min, max)	65 (42–83)	61 (35–84)	0.914
Tumor size (cm)	Median (min, max)	3 (1.2-6.5)	3.3 (1.2-10)	0.6526
pT classification (n = 97)	pT1-2	3 (4.5)	7 (23.3)	0.0089
	pT3-4	64 (95.5)	23 (76.7)	
Tumor grade (n = 98)	G1-2	8 (11.8)	3 (10.0)	1.0
	G3	60 (88.2)	27 (90.0)	
pN classification (n = 97)	pN0	12 (17.9)	5 (16.7)	1.0
	pN1-2	55 (82.1)	25 (83.3)	
pM classification (n = 97)	pM0	62 (92.5)	27 (90.0)	0.6996
	pM1	5 (7.5)	3 (10.0)	
Pn classification (n = 96)	Pn0	0 (0.0)	1 (3.5)	0.3021
	Pn1	67 (100.0)	28 (96.6)	
Lymphatic invasion (n = 97)	Negative	13 (19.4)	6 (200.)	1.0
	Positive	54 (80.6)	24 (80.0)	
Venous invasion (n = 97)	Negative	53 (79.1)	22 (73.3)	0.6025
	Positive	14 (20.9)	8 (26.7)	
R classification (n = 97)	R0	45 (67.2)	22 (73.3)	0.6382
	R1	22 (32.8)	8 (26.7)	
Adjuvant chemo (n = 89)	Untreated	1 (1.6)	2 (7.4)	0.2174
	Treated	61 (98.4)	25 (92.6)	
Tumor budding 10-in-10	Low-grade	16 (26.2)	10 (33.3)	0.6221
(n = 91)	High-grade	45 (73.8)	20 (66.7)	
Intratumoral budding	Negative/Low	26 (38.2)	12 (41.4)	0.8224
(n = 97)	Medium/High	42 (61.8)	17 (58.6)	
°Fisher’s Exact test				

Cutoff 10%.

## Discussion

This study is presenting a novel approach concerning the analysis of RKIP expression in pancreatic cancer, considering not only the expression pattern in the main tumor body but also in the tumor buds, in association with tumor and patient characteristics, in a well characterized cohort of 114 PDAC patients with known follow-up and adjuvant therapy information. The present study expands our understanding on the role of RKIP in pancreatic cancer on several aspects. First of all, we found a significant progressive loss of RKIP expression between normal pancreatic ductal epithelia (average: 73.76%), precursor lesions (PanINs; average: 36.71%), pancreatic cancer (average 20%) and matched lymph node metastases (average 8.3%; p < 0.0001). This implies that progressive RKIP loss may play a key role in the pancreatic neoplastic transformation and that it is taking place even before invasion occurs. Similar findings were observed in a previous study concerning esophageal Barrett mucosa, where a down-regulation of RKIP was found in high-grade dysplasia compared with non-dysplastic lesions [32]. Our findings are partially in keeping with previous studies on RKIP expression on pancreatic cancer, which have also demonstrated a significant RKIP loss in cancer tissues compared with normal pancreatic tissue and an association of RKIP loss with presence of nodal and/or distant metastases as well as reduced patient survival [33,34]. In our cohort, however, no significant correlation of RKIP loss with nodal (pN stage) or distant metastases (pM stage) was found. This could be due to differences in the patient cohorts, the different immunohistochemical methods and assessment protocols as well as different cut-offs used.

RKIP has been found to play a major role in the regulation of EMT by intervening in Raf-1/MEK/ERK and NF-kB mediated signaling [3-6]. NF-kb activation and subsequent activation of its downstream transcriptional target SNAIL induces EMT through down-regulation of E-cadherin and negatively regulates RKIP in cancer cells [35]. Moreover, RKIP loss enhances cellular motility by inducing the expression/stabilization of β-catenin, vimentin, MET and PAK1 [36] and augments oxidative stress–mediated activation of the p38 mitogen activated protein kinase, which, in turn, inactivates Glycogen synthase kinase-3b (GSK3b) by phosphorylating it at the inhibitory T390 residue. This pathway de-represses GSK3b inhibition of oncogenic substrates causing stabilization of cyclin D, which induces cell-cycle progression and β-catenin, SNAIL, and SLUG, which promote EMT [37]. In keeping with this, our study demonstrates a strong correlation of the RKIP loss with morphological hallmarks of EMT, like high-grade tumor budding at the invasive tumor front (p = 0.0048). The association of RKIP loss with high-grade budding remained strong when analyzing intratumoral budding, occuring within the main tumor body (p = 0.0373).

Further, we identified a variation of RKIP protein expression between the main tumor body and the tumor buds. RKIP expression was found to be significantly and consistently lower in the tumor buds (average 6%) compared to the main tumor body (average 20%; p < 0.005). Moreover, after using a matched pairs analysis a statistically significant loss of RKIP expression (p < 0.0001) was observed between the tumor and corresponding tumor buds. Our results suggest that this variation in the localisation of RKIP loss within the cancer tissue is relevant for the association with specific aggressive phenotypic features. RKIP loss in the main tumor body was marginally associated with higher T-stage (0.0599) as well as with the presence of peritumoral (0.0048) and intratumoral budding (p = 0.0373), while RKIP loss in the tumor buds exhibited a significant association with increased T stage (p = 0.0089). This finding underlines the predictive role of RKIP loss in the main tumor body concerning the presence of high-grade budding throughout the tumor. Additional RKIP loss in the tumor buds themselves seems to be involved in the propagation of the neoplastic process and to lead to increased aggressiveness of the neoplasm.

RKIP, by inhibiting the Raf-MEK-ERK, NFκB, GRK and activating the GSK3β signaling pathways is implicated in the sensitization of cells to therapeutic drugs. When its expression is reduced or lost this could lead to significant resistance to cancer therapy [38]. However, unlike other tumor types, like colorectal cancer, we could not identify any association between loss of RKIP and treatment response in our pancreatic cancer cohort. Because of the small number of untreated patients in this study (only 3 patients) no statistical comparison between treated and untreated patients could be performed.

Our present results should be understood in the context of the study limitations. Although TMAs provide an efficient and cost-effective tool for testing a comprehensive panel of potential biomarkers on a large number of tumor specimens, the TMA technique could raise concerns related to the sampling of large, heterogeneous tumors. The effect of tumor heterogeneity was minimized by sampling at least two punches from the center and two from the invasive front and evaluating the average protein expression across the total number of samples. Our study may further be limited by the relatively small number of PDAC patients and the fact that all cases come from a single center. Nonetheless, our study benefits from complete clinicopathological data with information on adjuvant therapy and follow-up and the adherence to the REMARK guidelines which are essential for proposing prognostic biomarkers.

In conclusion, our findings suggest that progressive loss of RKIP may play a key role in the neoplastic transformation of pancreas and correlates with aggressive features of PDAC. Moreover, RKIP loss seems to be strongly associated with EMT in pancreatic cancer, as reflected by the presence of high-grade tumor budding. Further characterization of the budding cells is needed in order to identify a “budding-promoting profile” and to underline the similarities between budding cells and EMT process in pancreatic cancer.

## Competing interests

The authors declare that they have no competing interests.

## Authors’ contributions

EK has made substantial contributions to conception and design, to acquisition and interpretation of data and has drafted the manuscript. IZ performed the statistical analysis and has been involved in revising the manuscript critically for important intellectual content. BG has been involved in revising the manuscript critically for important intellectual content. AKP has made substantial contributions to acquisition of data and has been involved in revising the manuscript critically for important intellectual content, AL participated in the design and coordination of the study, has been involved in revising the manuscript critically for important intellectual content and has given the final approval of the version to be published. AP participated in the design and coordination of the study, has been involved in revising the manuscript critically for important intellectual content and has given the final approval of the version to be published. All authors read and approved the final manuscript.
